# Quantification of SNAP-25 with mass spectrometry and Simoa: a method comparison in Alzheimer’s disease

**DOI:** 10.1186/s13195-022-01021-8

**Published:** 2022-06-04

**Authors:** Johanna Nilsson, Nicholas J. Ashton, Andrea L. Benedet, Laia Montoliu-Gaya, Johan Gobom, Tharick A. Pascoal, Mira Chamoun, Erik Portelius, Andreas Jeromin, Muriel Mendes, Henrik Zetterberg, Pedro Rosa-Neto, Ann Brinkmalm, Kaj Blennow

**Affiliations:** 1 Department of Psychiatry and Neurochemistry, Institute of Neuroscience and Physiology, the Sahlgrenska Academy at the University of Gothenburg, SE-43180 Mölndal, Gothenburg, Sweden; 2 Wallenberg Centre for Molecular and Translational Medicine, University of Gothenburg, Gothenburg, Sweden; 3 Department of Old Age Psychiatry, Maurice Wohl Clinical Neuroscience Institute, King’s College London, London, UK; 4 NIHR Biomedical Research Centre for Mental Health & Biomedical Research Unit for Dementia at South London & Maudsley NHS Foundation, London, UK; 5 Translational Neuroimaging Laboratory, McGill University Research Centre for Studies in Aging, Alzheimer’s Disease Research Unit, Douglas Research Institute, Le Centre intégré universitaire de santé et de services sociaux (CIUSSS) de l’Ouest-de-l’Île-de-Montréal, Montreal, Canada; 6 Department of Neurology and Neurosurgery, Psychiatry and Pharmacology and Therapeutics, McGill University, Montréal, Canada; 7 Clinical Neurochemistry Laboratory, Sahlgrenska University Hospital, SE-43180 Mölndal, Sweden; 8 Quanterix. Corp, Billerica, MA USA; 9 UK Dementia Research Institute at UCL, London, UK; 10 Department of Neurodegenerative Disease, UCL Institute of Neurology, London, UK; 11 Hong Kong Center for Neurodegenerative Diseases, Hong Kong, China; 12 Montreal Neurological Institute, Montréal, QC Canada

**Keywords:** Alzheimer’s disease, Synaptic pathology, Mass spectrometry, Biomarkers, SNAP-25

## Abstract

**Background:**

Synaptic dysfunction and degeneration are central to Alzheimer’s disease (AD) and have been found to correlate strongly with cognitive decline. Thus, studying cerebrospinal fluid (CSF) biomarkers reflecting synaptic degeneration, such as the presynaptic protein synaptosomal-associated protein 25 (SNAP-25), is of importance to better understand the AD pathophysiology.

**Methods:**

We compared a newly developed Single molecule array (Simoa) immunoassay for SNAP-25 with an in-house immunoprecipitation mass spectrometry (IP-MS) method in a well-characterized clinical cohort (*n* = 70) consisting of cognitively unimpaired (CU) and cognitively impaired (CI) individuals with and without Aβ pathology (Aβ+ and Aβ−).

**Results:**

A strong correlation (Spearman’s rank correlation coefficient (*r*_s_) > 0.88; *p* < 0.0001) was found between the Simoa and IP-MS methods, and no statistically significant difference was found for their clinical performance to identify AD pathophysiology in the form of Aβ pathology. Increased CSF SNAP-25 levels in CI Aβ+ compared with CU Aβ− (Simoa, *p* ≤ 0.01; IP-MS, *p* ≤ 0.05) and CI Aβ− (Simoa, *p* ≤ 0.01; IP-MS, *p* ≤ 0.05) were observed. In independent blood samples (*n* = 32), the Simoa SNAP-25 assay was found to lack analytical sensitivity for quantification of SNAP-25 in plasma.

**Conclusions:**

These results indicate that the Simoa SNAP-25 method can be used interchangeably with the IP-MS method for the quantification of SNAP-25 in CSF. Additionally, these results confirm that CSF SNAP-25 is increased in relation to amyloid pathology in the AD continuum.

**Supplementary Information:**

The online version contains supplementary material available at 10.1186/s13195-022-01021-8.

## Background

Synaptic degeneration and dysfunction are hallmarks of several neurodegenerative diseases, with Alzheimer’s disease (AD) being the most widely studied [1]. In AD, synaptic degeneration has been found to correlate strongly with cognitive decline, even more so than amyloid-beta (Aβ) plaque pathology [2]. The major role of synaptic dysfunction in AD pathophysiology, combined with the significant association of synaptic degeneration in relation to cognitive change, makes an argument for implementing synaptic biomarkers in the routine assessment of AD, which may be useful for diagnostics, disease staging, and prediction of progression. Furthermore, synaptic biomarkers have the potential to serve as tools to monitor downstream effects on synaptic function and integrity of treatments in drug trials.

**Fig. 1:**
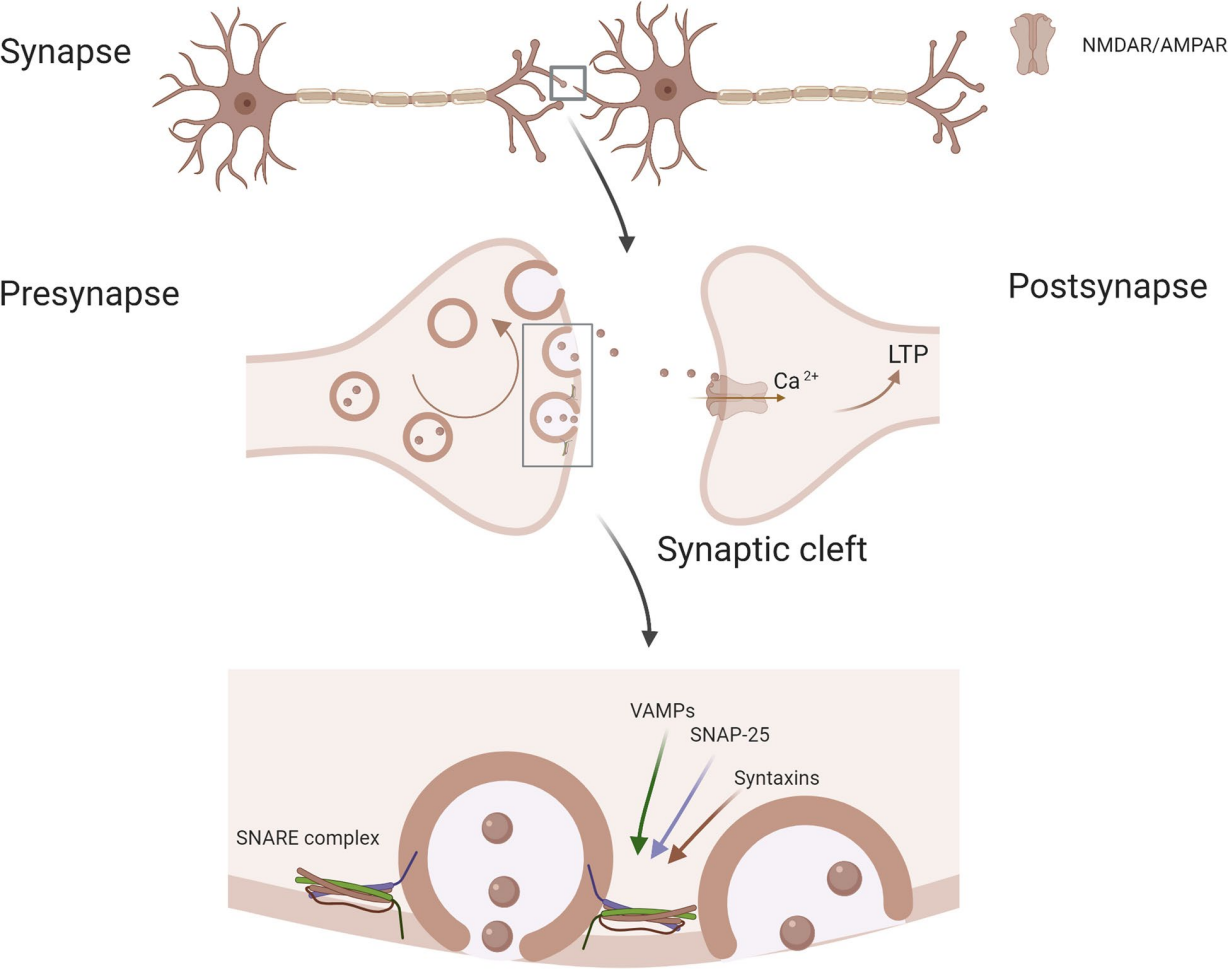
Schematic illustration of SNAP-25, its cellular location, and synaptic processes it partakes in. Created with BioRender.com

Synaptosomal-associated protein 25 (SNAP-25) is a presynaptic soluble N-ethylmaleimide-sensitive factor attachment receptor (SNARE) protein essential for cognitive function due to its key role in vesicular exocytosis. Together with vesicle-associated membrane proteins (VAMP) and syntaxins, it forms SNARE complexes, allowing for Ca^2+^-triggered vesicle fusion by mediation of the junction of synaptic vesicles to the presynaptic membrane (Fig. 1) [3]. SNAP-25 was first detected in CSF in 1999 [4] and was later confirmed to be decreased in AD brain tissue [5]. Since then, both mass spectrometry (MS) and conventional immunoassay methods, such as enzyme-linked immunosorbent assays (ELISA), have been developed for its quantification [5–8]. A number of studies have found that CSF levels of SNAP-25 are significantly higher in AD [5–8] and in Creutzfeldt-Jakob disease [9], in comparison to healthy age-matched controls. However, developing evidence suggests that SNAP-25, amongst other synaptic biomarkers, is specifically associated with amyloid pathology, as no change has been found in SNAP-25 levels in non-amyloid pathologies, such as in frontotemporal dementia (FTD) [10]. In support of this idea, SNAP-25 is significantly elevated in cognitively unimpaired (CU) individuals with detectable amyloid pathology compared to those without [11]. Despite strong genetic associations, limited success in differentiating healthy controls from psychiatric disorders, such as bipolar disorder (no change) or schizophrenia (small increase), using SNAP-25 CSF levels has been observed [12, 13].

MS is commonly used as the gold standard for biomarker quantification. The technique has high specificity, reproducibility, and sensitivity, and furthermore, it is also very versatile allowing for the quantification of multiple proteins or protein isoforms in a single analysis run. Other common techniques for biomarker quantification, guided by preliminary MS studies, include immunoassays, which, especially in the era of ultrasensitive semi-automated methods (e.g., Single molecule array (Simoa)), have high-throughput and high analytical sensitivity. This is of particular importance when wanting to quantify neurodegenerative processes in blood [14].

In this study, we compared a newly developed Simoa assay for SNAP-25 with our in-house developed IP-MS method in a well-characterized clinical cohort containing cognitively unimpaired (CU) and cognitively impaired (CI) patients with and without Aβ pathology. We further aimed to investigate if the Simoa assay can detect SNAP-25 in plasma.

## Method

### Study design and population

This study was performed in two stages. First, the CSF analysis included the quantification of SNAP-25 with both Simoa and IP-MS in a subset of participants (*n* = 71) from the Translational Biomarkers in Aging and Dementia cohort (TRIAD, McGill University, Canada) [15]. The participants were classified according to cognitive status, as impaired (CI) and unimpaired (CU), as well as according to amyloid status, positive (+) and negative (−), resulting in four groups: CU−, CU+, CI−, and CI+. In addition, subjects younger than 25 years old were grouped separately as “Young.” The participants which were classified as CI either had a clinical diagnosis of AD, mild cognitive impairment (MCI), and FTD or had an unconfirmed diagnosis together with a Clinical Dementia Rating (CDR) score greater than or equal to 0.5. In this cohort, amyloid positivity was indicated by CSF Aβ_1-42/40_ ≤ 0.068 [16]. The diagnosis of AD dementia followed the criteria for probable AD as recommended by the National Institute on Aging and the Alzheimer’s Association, with a CDR greater than 1. The MCI participants had a CDR of 0.5, subjective and objective memory impairments, and essentially normal activities of daily living while CU individuals were required to have a CDR of 0. FTD had a clinical diagnosis of the behavioral or semantic variant, had a CDR score greater than or equal to 0.5, and were Aβ negative. For TRIAD, all participants have provided written informed consent and the study has been approved by McGill University Institutional Review Board. The demographics of the cohort are shown group-wise in Table 1.

**Table 1:** Cohort demographics

Cohort	Group	***n*** (F/M)	Age	MMSE	Aβ_**1-42/1-40**_	P-tau_**181**_	T-tau
*TRIAD CSF*	*Young*	5 (3/2)	23 (1, 22–24)^a,b,c,d^	30 (0, 30–30)	0.095 (0.010, 0.088–0.10)^b,d^	24 (7, 23–31)^b^	191 (36, 161–222)^b^
	*CU−*	15 (11/4)	71 (5, 68–74)^a^	29 (1, 28–30)	0.093 (0.010, 0.082–0.098)^b,d^	33 (10, 29–42)^b^	252 (72, 199–335)^b^
	*CU+*^*6*^	10 (5/5)	73 (3, 70–76)^a^	29 (1, 29–30)	0.059 (0.011, 0.046–0.067)^a,b^	52 (22, 41–72)^b^	395 (126, 292–488)^b^
	*CI−*	12 (6/6)	65 (8, 60–68)	29 (3, 26–30)	0.095 (0.014, 0.091–0.099)^b^	33 (13, 19–36)^b^	250 (88, 159–294)^b^
	*CI+*^*8*^	28 (11/17)	68 (9, 60–77)	25 (4, 21–29)	0.042 (0.009, 0.037–0.051)	82 (50, 64–118)	551 (323, 398–778)

Notes: Detailed diagnosis for the CI+ group consisted of the following: 15 AD and 13 MCI, while the CI− group consisted of 3 MCI, 4 FTD, and 5 patients with uncertain diagnosis. Analysis of variance (ANOVA) followed by Tukey’s post hoc test was used for the continuous variables. The level of significance used was *p* ≤ 0.05. Data presented as median (standard deviation, interquartile interval) and in ng/L. ^a^Significant ANOVA post hoc Tukey test compared to CI−. ^b^Significant ANOVA post hoc Tukey test compared to CI+. ^c^Significant ANOVA post hoc Tukey test compared to CU−. ^d^Significant ANOVA post hoc Tukey test compared to CU+

*Abbreviations*: *MMSE* Mini-Mental State Exam Score, *Aβ*_*1-42/1-40*_ amyloid beta protein ratio 1-42/1-40, *P-tau*_*181*_ total tau, *T-tau* phosphorylated tau at amino acid Thr181, *CU−* cognitively unimpaired Aβ negative, *CU+* cognitively unimpaired Aβ positive, *CI−* cognitively impaired Aβ negative, *CI+* cognitively impaired Aβ positive

Second, the ability of the Simoa assay to detect SNAP-25 in plasma was evaluated in de-identified samples (*n* = 32) from patients > 80 years old collected at the Clinical Neurochemistry Laboratory, Mölndal, Sweden. The samples additionally underwent plasma p-tau_181_ and p-tau_231_ analysis to correlate with known blood markers of AD pathology. The Ethics Committee at the University of Gothenburg has approved the use of the samples (EPN 140811). Demographic information for the plasma samples is detailed in Suppl. Table 1.

### SNAP-25 analysis

**Fig. 2:**
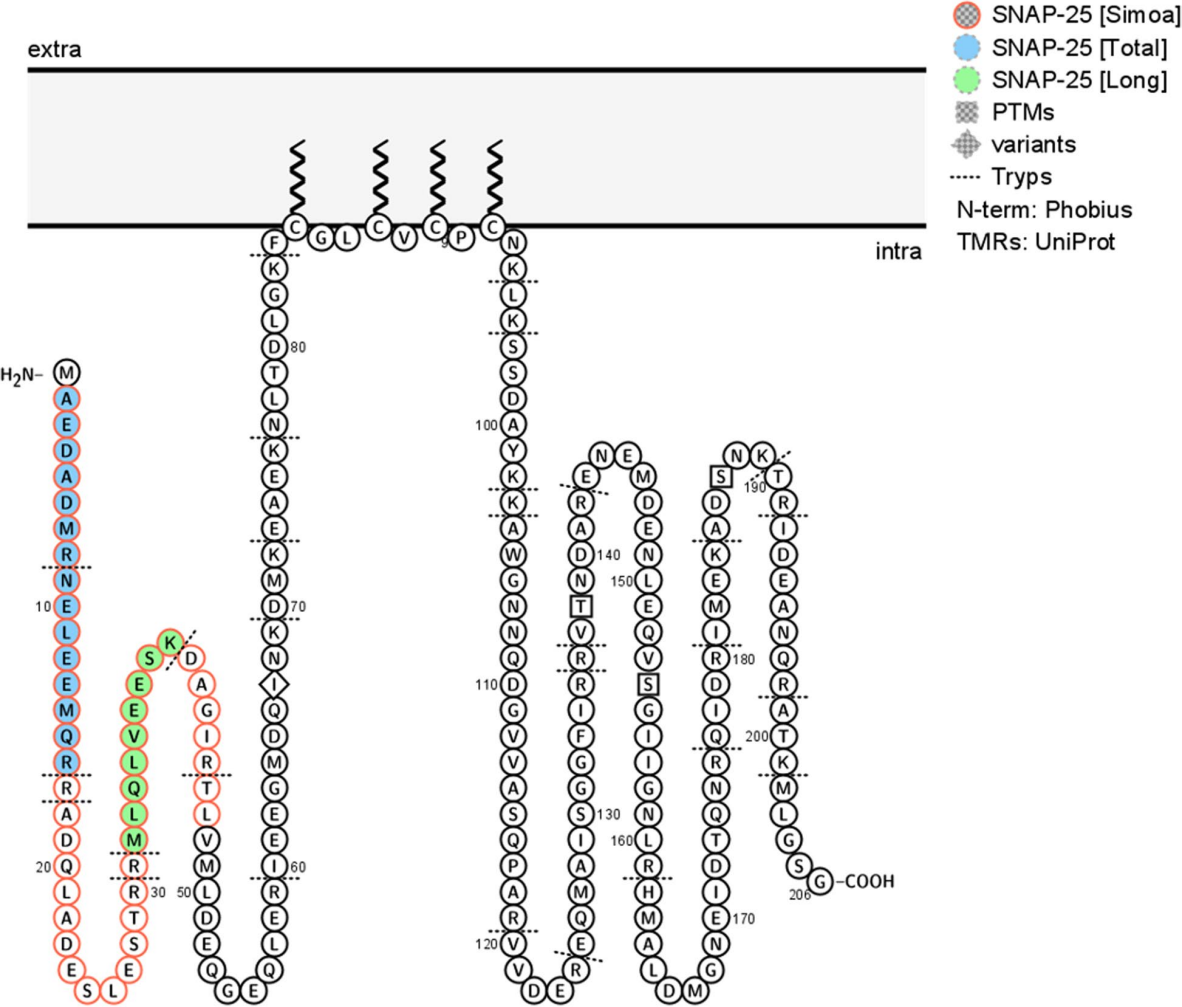
Schematic illustration of the synaptic protein SNAP-25 protein sequence and membrane attachment, including the two peptides targeted by IP-MS, SNAP-25 [Long] (fill color green) and SNAP-25 [Total] (fill color blue), and the region targeted by the SNAP-25 [Simoa] (frame color pink). Created with Protter [17]

The CSF samples were collected by lumbar puncture, transferred to polypropylene tubes for centrifugation (2200 × g for 10 min, 20°C), and permanently stored at −80°C in 1-mL aliquots pending analyses. For the plasma samples, whole blood was collected into EDTA-treated tubes, centrifuged (4000 × g for 10 min), the supernatant removed, and permanently stored at −80°C in 1-mL aliquots pending analyses. SNAP-25 was measured according to the manufacturer’s instructions on the HD-X instrument (Quanterix). EDTA plasma was analyzed at the same volume both neat and a 2× dilution using the same protocol. The assay targets the soluble N-terminal fragment of SNAP-25, specifically the amino acids 2-47 (Fig. 2). The quantification of SNAP-25 by IP-MS was according to our in-house method, as described previously [5, 18, 19]. The IP-MS method quantifies two SNAP-25 peptides hereafter termed SNAP-25 [Long] (amino acids 32-40) and SNAP-25 [Total] (N-terminally acetylated amino acids 2-16) (Fig. 2). The selection of the SNAP-25 peptides is based on previously published work where Ac-2-16 [Total] is found to be present in all identified soluble SNAP-25 forms while 32-40 [Long] is present only in the longest soluble forms Ac-2-46 and Ac-2-47 [5]. Briefly, 200 μL of CSF was immunoprecipitated using the mouse monoclonal antibody SM181 (0.5 μg per sample, Nordic BioSite) which targets N-terminally acetylated SNAP-25 amino acid 1-11 and IgG-coated magnetic beads Dynabeads M-280 Sheep anti-mouse or anti-rabbit IgG, Thermo Fisher Scientific on the KingFisher™ Flex System (Thermo Fisher Scientific). The samples were then digested using trypsin/Lys C (0.4 μg per sample, Promega Co) overnight at 37°C and heavy isotope-labeled standards (AQUA QuantProHeavy; Thermo Fisher Scientific) were added. Quantification was then performed with liquid chromatography/high-resolution parallel reaction monitoring (PRM) performed on a Q Exactive quadrupole-orbitrap mass spectrometer coupled to an Ultimate 3000 chromatography system (Thermo Fisher Scientific) equipped with a Hypersil Gold reversed-phase column (dim. 100×2.1 mm, particle size 1.9 μm). Details of LC-MS/MS settings can be found in Suppl. Table 2.

### Data processing and statistical analysis

Data processing of MS raw data was performed in Pinpoint 1.3.0 (Thermo Fisher Scientific). The complete peak area was determined after using four points of smoothing. The detected fragment ion peaks were manually inspected for accuracy and absence of interferences from other peptides than the peptide of interest, including fragments originating from other product ions in the same pair/triplet. Statistical analysis was performed in GraphPad Prism 8.3.0 (GrahpPad Software, Inc.). Analysis of variance (ANOVA) followed by Tukey’s post hoc test was used to compare demographic continuous variables between groups. Spearman rank correlation analysis was used to test the correlation between continuous variables. To evaluate the biomarker performance, group-wise comparisons were assessed using Kruskal-Wallis with post hoc Dunn’s test and false discovery rate (FDR) correction for multiple comparisons using the two-stage step-up method of Benjamini, Krieger, and Yekutieli (*Q* = 0.05). The area under the curve (AUC) was provided by the receiver operating characteristic curve (ROC) contrasting groups. To compare the AUC values head-to-head, the DeLong test was used in the MedCalc statistical software (MedCalc Software Ltd.). Fold changes were calculated by dividing the SNAP-25 concentrations by the median SNAP-25 concentration of the comparing group. Kruskal-Wallis with post hoc Dunn’s test and false discovery rate (FDR) correction for multiple comparisons using the two-stage step-up method of Benjamini, Krieger, and Yekutieli (*Q* = 0.05) was used to compare fold changes between the biomarkers. No statistical correction for age or gender was performed.

## Results

### Validation of the SNAP-25 Simoa assay and the SNAP-25 MS assay

We performed an independent partial validation (parallelism, spike recovery, and lower limit of quantification (LLOQ)) of the Simoa SNAP-25 assay for its use in CSF. The Simoa assay demonstrated good parallelism (10.9–14.1%; Suppl. Table 3), and also good recovery (88.4–104.3%), when spiking recombinant SNAP-25 into CSF samples with different native SNAP-25 concentrations (Suppl. Table 4). The functional LLOQ was determined as 16.1 pg/mL (Suppl. Table 5).

We have earlier performed an independent partial validation (precision and measurement range (LLOQ and upper limit of quantification (ULOQ))) of the MS SNAP-25 assay for its use in CSF. The MS assay demonstrated good precision (Suppl. Table 7) for both peptides in the form of repeatability (6.5–13%) and within-lab reproducibility (13–22%). The LLOQ and ULOQ for SNAP-25 [Total] were determined to be 13 pmol/L and 1500 pmol/L, respectively. For SNAP-25 [Long], LLOQ and ULOQ were found to be 3 pmol/L and 900 pmol/L, respectively. Both validations are described in detail in supplementary appendix and no CSF sample in this study was found to be under the LLOQ of each respective method.

### Correlations between Simoa and MS SNAP-25 assays

**Fig. 3:**
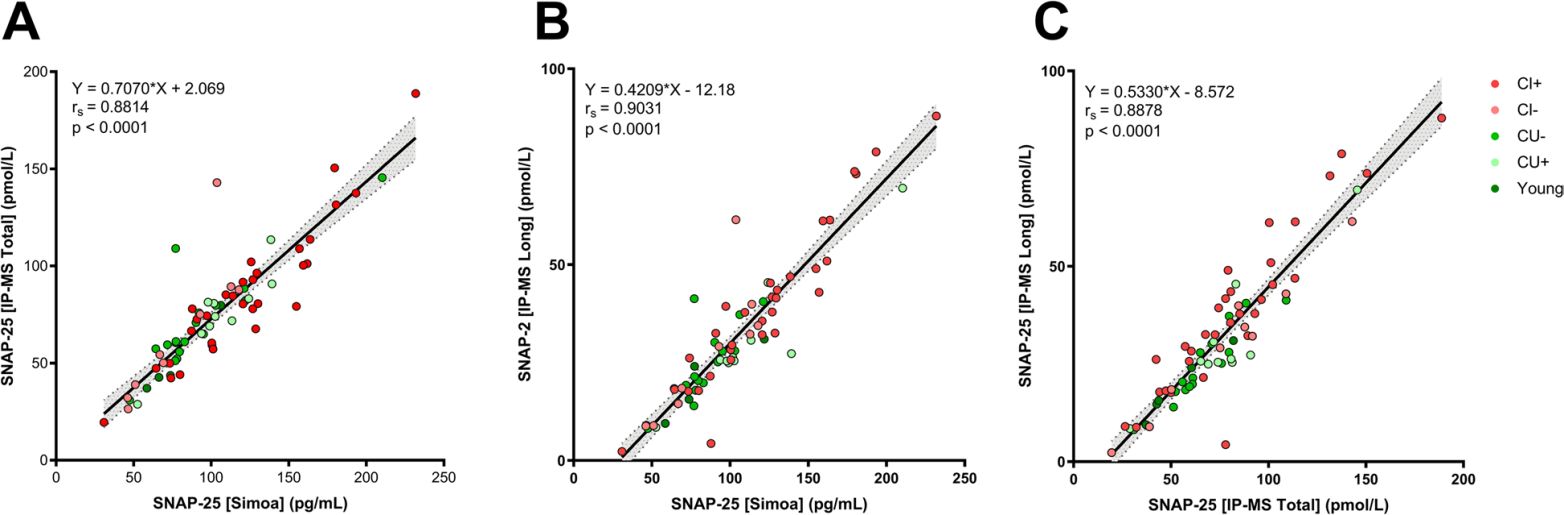
Correlations in the clinical CSF cohort consisting of young (*n* = 5), cognitively unimpaired Aβ negative (CU−, *n* = 15), cognitively unimpaired Aβ positive (CU+, *n* = 10), cognitively impaired Aβ negative (CI−, *n* = 12), and cognitively impaired Aβ positive (CI+, *n* = 28) between the two SNAP-25 forms, SNAP-25 [Long] and SNAP-25 [Total], quantified with MS and SNAP-25 [Simoa] with equation of slope, Spearman’s rank correlation coefficient (*r*_s_) and *p*-value. **A** SNAP-25 [Total] versus SNAP-25 [Simoa], **B** SNAP-25 [Long] versus SNAP-25 [Simoa], and **C** SNAP-25 [Total] versus SNAP-25 [Long]

The CSF SNAP-25 measurements on the two platforms showed a strong correlation: SNAP-25 [Simoa] versus SNAP-25 [Long]: Spearman’s rank correlation coefficient (*r*_s_) = 0.9031, 95% confidence interval (CI_95%_) = 0.85–0.94 *p* < 0.0001, Fig. 3A; SNAP-25 [Simoa] versus SNAP-25 [Total]: *r*_s_ = 0.8814, CI_95%_ = 0.81–0.93 *p* < 0.0001, Fig. 3B. The two MS peptides, SNAP-25 [Total] and SNAP-25 [Long], also correlated well with each other *r*_s_ = 0.8878, CI_95%_ = 0.82–0.93 *p* < 0.0001 (Fig. 3C).

### Diagnostic performance

**Fig. 4:**
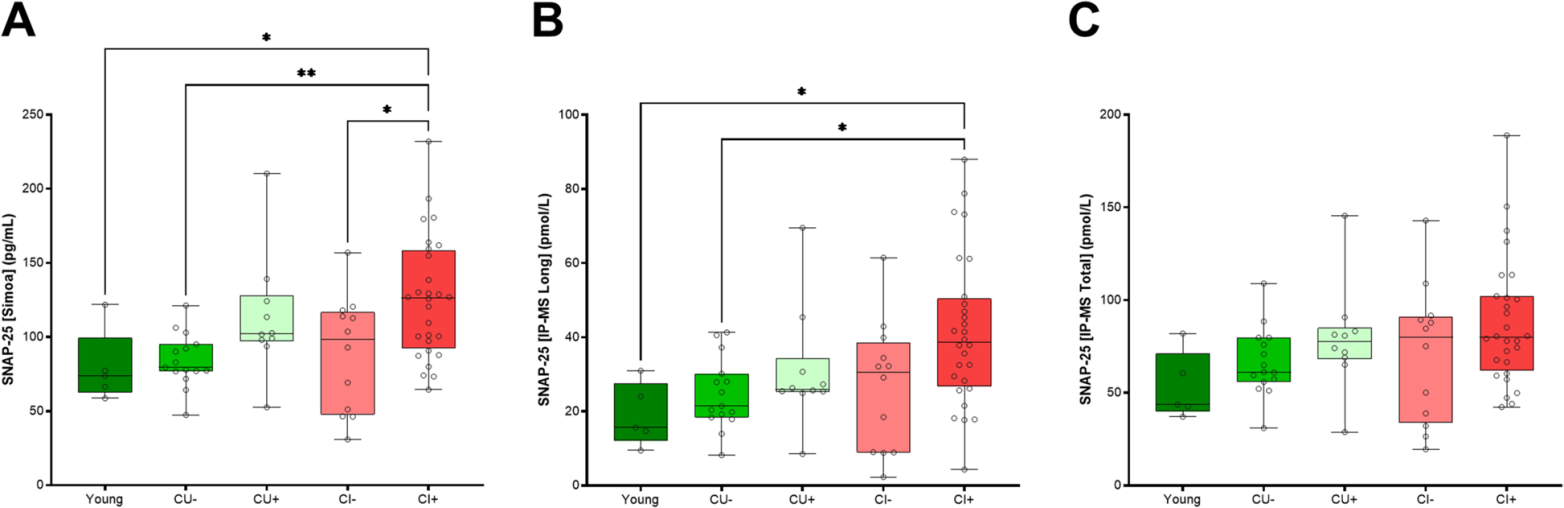
CSF SNAP-25 concentrations using the two platforms, SNAP-25 [Simoa] (**A**) and MS (SNAP-25 [Long] (**B**) and SNAP-25 [Total] (**C**) in the cohort consisting of young (*n* = 5), cognitively unimpaired Aβ negative (CU−, *n* = 15), cognitively unimpaired Aβ positive (CU+, *n* = 10), cognitively impaired Aβ negative (CI−, *n* = 12), and cognitively impaired Aβ positive (CI+, *n* = 28). Statistical comparison was performed with Kruskal-Wallis with post hoc Dunn’s test and false discovery rate (FDR) correction for multiple comparisons using the two-stage step-up method of Benjamini, Krieger, and Yekutieli (*Q* = 0.05). *p*-values: * *p* ≤ 0.05 and ** *p* ≤ 0.01. The bars indicate median with interquartile range

The two platforms, Simoa and IP-MS, showed a similar diagnostic performance and pattern. Both the SNAP-25 [Simoa] and the SNAP-25 [Long] (Fig. 4) showed significant increases for the CI+ group compared to CU− (SNAP-25 [Simoa], *p* = 0.0053; SNAP-25 [Long], *p* = 0.0233) and to the CI- (SNAP-25 [Simoa], *p* = 0.0302; SNAP-25 [Long], *p* = 0.0233) groups. No significant differences were found for the other groups with the exception for SNAP-25 [Simoa] which showed significant increases for the CI+ group compared to young (*p* = 0.0302). Despite also demonstrating mean increases, these significant differences were not found for the SNAP-25 [Total]. Comparing fold changes between the three measurements in the same diagnostic groups, no significant differences were found (Table 2).

**Table 2:** Receiver operating characteristic analysis and fold change results for the CSF SNAP-25 analysis on the two platforms

		CU− vs CU+	CI− vs CI+	CU− vs CI+	CU− vs CI−
SNAP-25 [Simoa]	***ROC***				
	*AUC (CI*^*7*^*)*	*0.79 (0.60–0.99)*	*0.74 (0.57–0.90)*	*0.82 (0.70–0.94)*	*0.54 (0.29–0.80)*
	*p-value*	0.0147*	0.0182*	0.0006***	*0.6063*
	***Fold change***				
	*Mean (Std)*	*1.42 (0.51)*	*1.39 (0.45)*	*1.57 (0.51)*	*1.1 (0.49)*
SNAP-25 [Long] IP-MS	***ROC***				
	*AUC (CI)*	*0.64*^*a*^ *(0.41–0.87)*	*0.70 (0.52–0.88)*	*0.77 (0.64–0.91)*	*0.54 (0.30–0.78)*
	*p-value*	*0.2441*	0.0448*	0.0034**	*0.7327*
	***Fold change***				
	*Mean (Std)*	*1.44 (0.76)*	*1.48 (0.73)*	*1.93 (0.94)*	*1.24 (0.81)*
SNAP-25 [Total] IP-MS	***ROC***				
	*AUC (CI)*	*0.71 (0.49–0.93)*	*0.61*^*a*^ *(0.41–0.82)*	*0.69*^*a*^ *(0.53–0.85)*	*0.53 (0.28–0.79)*
	*p-value*	*0.0855*	*0.2621*	0.0441*	*0.7697*
	***Fold change***				
	*Mean (Std)*	*1.29 (0.47)*	*1.21 (0.47)*	*1.43 (0.55)*	*1.15 (0.61)*

*Notes*: The DeLong test was used to statistically compare AUC values head-to-head. Kruskal-Wallis with post hoc Dunn’s test and false discovery rate (FDR) correction for multiple comparisons using the two-stage step-up method of Benjamini, Krieger, and Yekutieli (*Q* = 0.05) was used to compare fold changes in the same diagnostic groups between the biomarkers (no significance found). The level of significance used was *p* ≤ 0.05. ^a^Significant DeLong test compared to SNAP-25 Simoa (*p* ≤ 0.05)

*Abbreviations*: *CU−* cognitively unimpaired Aβ negative, *CU+* cognitively unimpaired Aβ positive, *CI−* cognitively impaired Aβ negative, *CI+* cognitively impaired Aβ positive, *ROC* receiver operating characteristic, *AUC* area under the curve, *CI* confidence interval, *Std* standard deviation

All three measurements showed a modest accuracy discriminating CI+ from CI− (SNAP-25 [Simoa], AUC = 0.74, CI_95%_ = 0.57–0.90; SNAP-25 [Total], AUC = 0.61, CI_95%_ = 0.41–0.82; SNAP-25 [Long], AUC = 0.70, CI_95%_ = 0.52–0.88). In comparing CI+ from CU−, SNAP-25 [Simoa] again had a higher AUC (AUC = 0.82, CI_95%_ = 0.70–0.94) in comparison to SNAP-25 [Total (AUC = 0.69, CI_95%_ = 0.53–0.85) and SNAP-25 [Long] (AUC = 0.77, CI_95%_ = 0.64–0.91) (Table 2). In both comparisons, SNAP-25 [Simoa] significantly outperformed (*DeLong*_Simoa vs Total, CI− vs CI+_ *p* = 0.0189 and *DeLong*_Simoa vs Total, CU− vs CI+_ *p* = 0.0121) the SNAP-25 [Total] but not the SNAP-25 [Long] (*DeLong*_Simoa vs Long, CI- vs CI+_ *p* = 0.7638 and *DeLong*_Simoa vs Total, CU- vs CI+_ *p* = 0.3040). A clear mean increase (fold change: 1.29–1.44) was observed for CU+ compared to CU− patients; however, this did not reach statistical significance likely due to the small sample size.

**Fig. 5:**
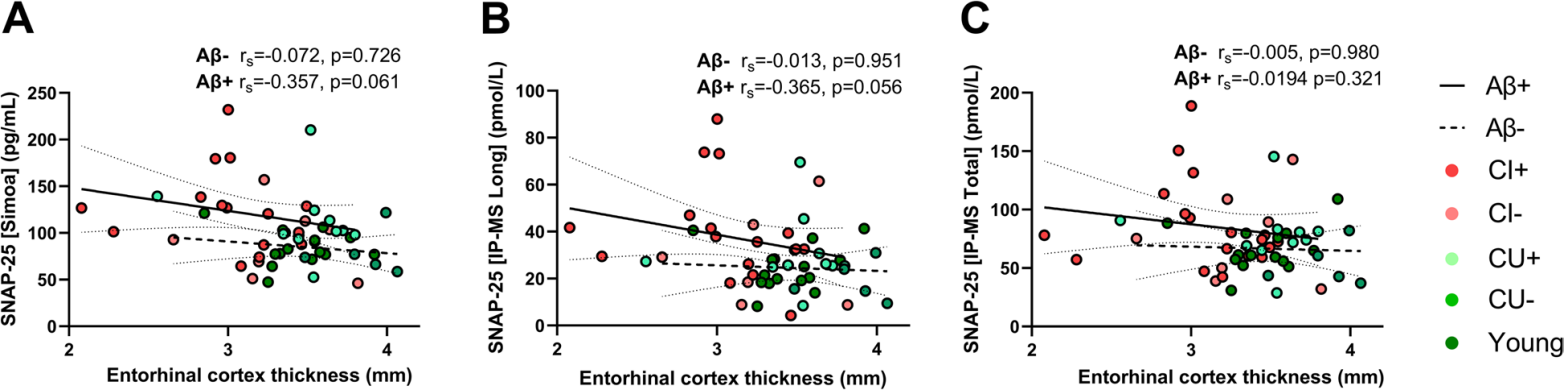
Associations of CSF SNAP-25 **A** [Simoa], **B** [Long], and **C** [Total] with entorhinal cortex thickness, divided by Aβ status with Spearman rank correlation coefficient and *p*-value

A moderate negative association with entorhinal cortex thickness, albeit not significant, was found in the Aβ+ group for both SNAP-25 [Simoa] (Fig. 5A, *r*_s_ = −0.357, *p* = 0.061) and for SNAP-25 [Long] (Fig. 5B, *r*_s_ = −0.365, *p* = 0.056). No association was found for SNAP-25 [Total] in the Aβ+ group (Fig. 5C), and in the Aβ− group, no associations at all were found for any of the SNAP-25 measurements. Additionally, no associations were found for hippocampal volume or cortical thickness in either group and for any of the SNAP-25 measurements (Suppl. Table 9).

### Plasma results

**Fig. 6:**
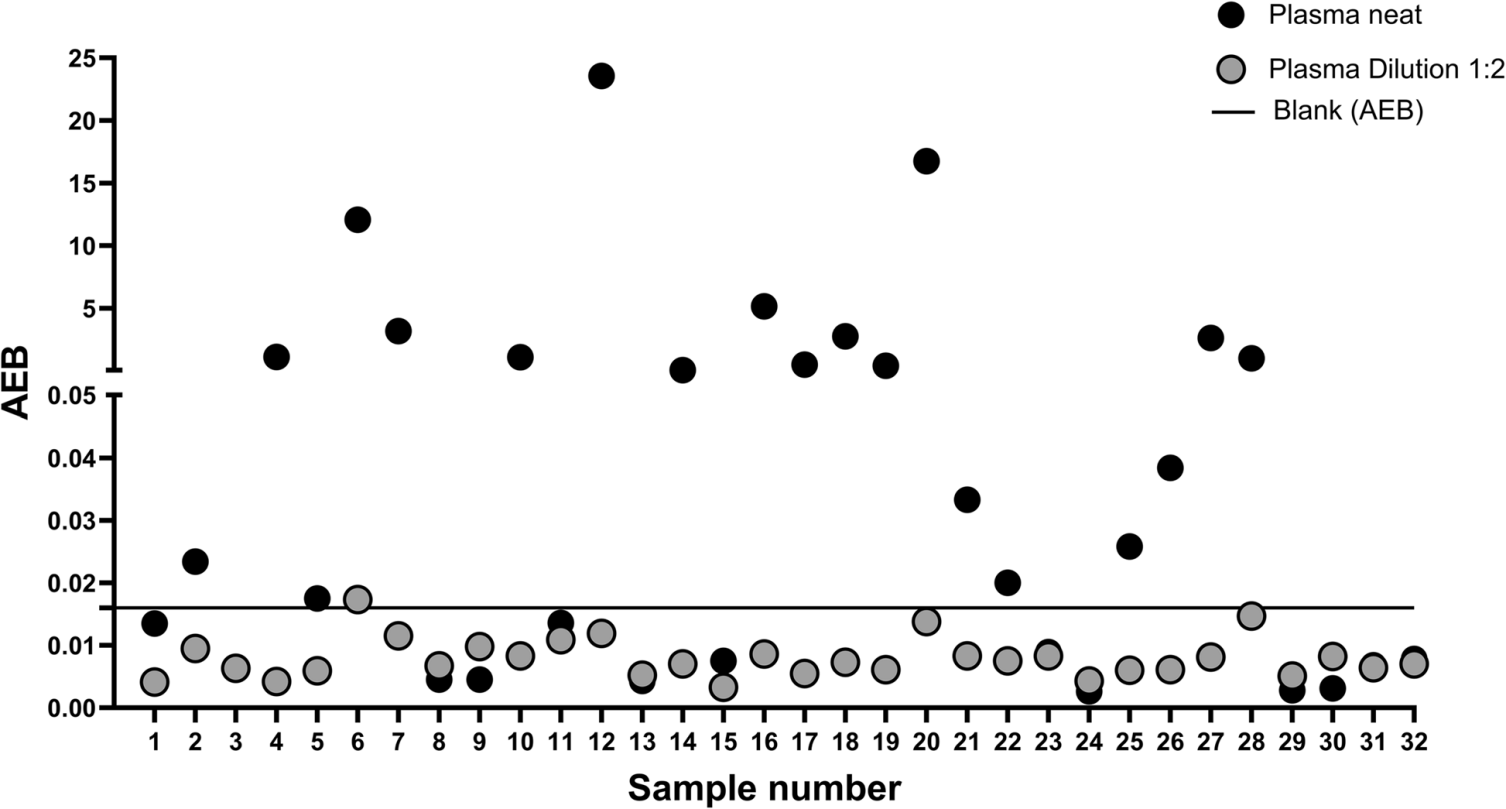
Average enzymes per bead (AEB) measurement for SNAP-25 [Simoa] in 32 plasma samples run in neat and 1:2 dilution

All plasma samples run at twofold dilution had an average enzyme per bead (AEB) below the background level of the Simoa assay (Fig. 6) and were thus below the limit of detection (LOD). When analyzed without dilution, several of the samples yielded high signals, above the LOD, however, did not follow a linear trend when diluted, indicating matrix effects and non-specific signal in the absence of sample diluent. The AEB signal of plasma SNAP-25 did not associate with established measures of AD pathology, P-tau_181_ and P-tau_231_, in the blood (Suppl. Table 10). The SNAP-25 assay was also utilized in order to try to detect plasma SNAP-25; however, no signal was observed in 0.5 mL of plasma (data not shown).

## Discussion

The presence of SNAP-25 in CSF was first demonstrated in the late 1990s [4], but it was not until advances in MS technologies and enrichment methods that allowed a detailed characterization of soluble forms of SNAP-25 that quantification in CSF from individual patients became possible [5]. As of today, SNAP-25 together with the presynaptic protein, growth-associated protein 43 (GAP-43), and the postsynaptic protein, neurogranin, are the most thoroughly investigated synaptic proteins in AD [5–8, 20–22]. Furthermore, CSF elevation of SNAP-25 has emerged as a potential biomarker of synaptic degeneration, in particular in AD. MS is often considered the gold standard of biomarker quantification, mainly because of its ability to identify and quantify specific protein forms with high specificity, reproducibility, and sensitivity. In this study, we describe a Simoa SNAP-25 method, which is semi-automated with a low sample volume requirement, and compare this immunoassay to our validated in-house IP-MS method.

In the comparison of the two platforms, we found very similar performances, namely the ability to distinguish Aβ+ from Aβ− individuals at preclinical and dementia stages and moderate fold changes (1.21–1.93). Furthermore, a strong correlation was found between the two platforms (*r*_s_ > 0.88) and when comparing SNAP-25 [Simoa] with SNAP-25 [Long - targeting only the SNAP-25 forms with the acetylated N-terminal and at least the first 40 amino acids], there was no statistical difference in either the ROC analysis or between their mean fold change. However, SNAP-25 [Total – targeting all SNAP-25 forms containing acetylated N-termini] did not perform as well as the SNAP-25 [Simoa] in separating Aβ+ from Aβ− individuals in the ROC analysis. This result is in agreement with an earlier study suggesting that the longer soluble forms of soluble SNAP-25, including the Ac-2-47 form targeted by both the Simoa and the IP-MS assays, provide the best differential diagnostic SNAP-25 biomarker of AD compared to controls [5]. To summarize, this suggests that the SNAP-25 Simoa method can be used interchangeably with the IP-MS method. In this study, SNAP-25 levels were increased in AD patients (CI Aβ+) in comparison with healthy controls (CU Aβ−) — which has been published repeatedly using both mass spectrometry and ELISA [5–8], and now also Simoa. SNAP-25, however, is less studied in patients with cognitive impairment without Aβ pathology, e.g., FTD, PSP, and CBD. The exception is one study which found similar levels of SNAP-25 in FTD compared to controls and significantly increased levels in AD in comparison with both the FTD and control group [10]. Our results support this, indicating that this is true even for a mixed group of non-AD dementias, represented by CI Aβ−. In addition, we also show evidence of SNAP-25 increasing at the preclinical stages, if Aβ pathology is present, although this did not reach statistical significance likely because of this comparison being underpowered.

Most studies on synaptic proteins and other potential biomarkers of neurodegenerative diseases focus on CSF as the sample matrix due to its proximity to the brain. However, much effort has been made in identifying AD in more accessible biofluids [23]. Blood, for example, is much more easily accessible and more feasible in large studies and for widespread clinical use. However, the total protein content of blood is significantly (hundreds-fold) higher than that of CSF while concurrently the target protein concentration originating from the central nervous system exists likely at femtomolar levels. Furthermore, depending on the target, peripheral expression can be another issue in the quantification of synaptic proteins as blood biomarkers. For example, neurogranin is also produced in high levels by red blood cells, making the quantification of neurogranin as a synaptic biomarker in blood not feasible [1]. However, for most proteins, the issue is to be able to detect them at all due to the low concentrations, which seems to be the case for SNAP-25. There is thus a clear need for methods that have a higher sensitivity than currently available technologies and allow for quantification at those low levels. The new automated ultrasensitive immunoassays such as the Simoa platform have been theorized and designed to be able to quantify several previously undetected blood biomarkers such as different forms of phosphorylated tau [24]. Yet, our results indicate that for SNAP-25 this is still not enough for its detection in plasma with the current assay. One study reported quantification of SNAP-25 in neuronally derived exosomes extracted from serum, indicating that there is a SNAP-25 pool present in blood [25]. Neuronally derived exosomes entail an opportunity of origin-specific biomarker quantification in a distant easy access matrix such as blood. Notwithstanding, presently, exosome extraction for biomarker quantification has methodological hurdles, in particular specificity, to overcome to be feasible for large-scale studies and clinical use [1, 26].

## Limitations

The strength of this study is that we compare the new Simoa method with the current gold standard, i.e., mass spectrometry. Furthermore, we used a clinical cohort with amyloid PET imaging to evaluate the two assays but with a limited number of samples which might have hampered our ability to detect minor differences in performance between the assays.

## Conclusions

To conclude, SNAP-25 is a synaptic protein with promising potential as a CSF biomarker of synaptic pathology in AD. In this study, we show that SNAP-25 quantification with the new Simoa assay strongly correlates with the SNAP-25 quantification performed with the current IP-MS method, meaning that the newly developed Simoa assay for the quantification of SNAP-25 can be used interchangeably with the current IP-MS method. This study demonstrates the importance of MS-based biomarker discovery and validation that utilizes highly specific quantification of multiple variants. This important step drives the development of new highly accurate methods, which increases the accessibility of biomarker quantification in larger research cohorts or ad hoc testing in clinical routine and therapeutic trials.

## Supplementary Information

**Additional file 1.** Supplementary tables.

## Data Availability

Derived data supporting the findings of this study are available from the corresponding author on request, providing data transfer is in agreement with the participating centre national legislation and institutional review centre.

## Abbreviations

AD: Alzheimer’s disease
AEB: Average enzyme per bead
Aβ: Amyloid beta
ANOVA: Analysis of variance
AUC: Area under the curve
Aβ_1-42_: Aβ peptide 1-42
CSF: Cerebrospinal fluid
CU: Cognitively unimpaired
CU Aβ+: Cognitively unimpaired Aβ negative
CU Aβ−: Cognitively unimpaired Aβ negative
CI: Cognitively impaired
CI_95%_: 95% confidence interval
CI Aβ−: Cognitively impaired Aβ negative
CI Aβ+: Cognitively impaired Aβ positive
CV: Coefficient of variation
ELISA: Enzyme-linked immunosorbent assay
FTD: Frontotemporal dementia
GAP-43: Growth-associated protein 43
IP-MS: Immunoprecipitation mass spectrometry
LLOQ: Lower limit of quantification
LOD: Limit of detection
MMSE: Mini-Mental State Exam
MS: Mass spectrometry
PRM: Parallel reaction monitoring
P-tau_181_: Tau phosphorylated at Thr181
P-tau_231_: Tau phosphorylated at Thr231
ROC: Receiver operating characteristic curve
r_s_: Spearman’s rank correlation coefficient
Simoa: Single molecule array
SNAP-25: Synaptosomal-associated protein 25
SNARE: Soluble N-ethylmaleimide-sensitive factor attachment receptor
T-tau: Total tau
ULOQ: Upper limit of quantification
VAMP: Vesicle-associated membrane protein
