# Supplementary material for: Quantification of SNAP-25 with mass spectrometry and Simoa: a method comparison in Alzheimer’s disease
Source: Alzheimers Res Ther. 2022 Jun 4;14:78. doi: 10.1186/s13195-022-01021-8 (PMC9166380; doi:10.1186/s13195-022-01021-8)
Supplement: Supplementary file 1 — Additional file 1. Supplementary tables. [file 13195_2022_1021_MOESM1_ESM.docx]

**APPENDIX**

**Supplementary Table 1**. Demographics of the measured plasma samples. ^1^ Phosphorylated tau at amino acid Thr181; ^2^Phosphorylated tau at amino acid Thr231.

|  | **Age** | **Sex** | **P-tau_181_^1^** | **P-tau_231_^2^** |
| --- | --- | --- | --- | --- |
| **1** | 93 | F | 15 | 18 |
| **2** | 88 | F | 11 | 12 |
| **3** | 87 | M | 11 | 11 |
| **4** | 88 | M | 9 | 9 |
| **5** | 84 | M |  |  |
| **6** | 83 | M | 9 | 9 |
| **7** | 84 | F | 11 | 10 |
| **8** | 91 | F | 11 | 12 |
| **9** | 91 | F | 8 | 7 |
| **10** | 82 | F | 8 | 8 |
| **11** | 84 | F | 17 | 20 |
| **12** | 84 | M | 10 | 10 |
| **13** | 91 | F | 10 | 11 |
| **14** | 86 | F | 7 | 7 |
| **15** | 88 | F | 10 | 9 |
| **16** | 92 | F | 12 | 11 |
| **17** | 82 | M | 9 | 10 |
| **18** | 85 | M | 7 | 6 |
| **19** | 83 | M | 8 | 7 |
| **20** | 90 | M | 12 | 12 |
| **21** | 85 | F | 35 | 19 |
| **22** | 91 | F | 9 | 10 |
| **23** | 87 | M |  |  |
| **24** | 87 | F | 11 | 10 |
| **25** | 94 | F | 7 | 6 |
| **26** | 82 | M | 13 | 12 |
| **27** | 88 | F | 6 | 8 |
| **28** | 85 | F | 11 | 12 |
| **29** | 91 | M | 10 | 9 |
| **30** | 88 | M | 5 | 4 |
| **31** | 84 | F | 6 | 8 |
| **32** | 89 | F | 9 | 11 |

**Supplementary Table 2. LC-MS/MS settings for the analysis of SNAP-25**.

|  | Parameter | Setting |
| --- | --- | --- |
| LC | Sample injection volume | 60 µL |
|  | Flow-rate | 0.3 mL/min |
|  | Gradient | Broken; 16–19%B (8 min), 19–30%B (2 min) |
|  | Total cycle time | 15 min |
|  | Mobile phase A | 0.1% formic acid in water (v/v) |
|  | Mobile phase B | 0.1% formic acid/84% acetonitrile in water (v/v) |
|  |  |  |
| Electrospray | Mode | Positive |
|  | Spray voltage | +4100 V |
|  | Capillary temperature | 320 °C |
|  | Sheath gas | 25 |
|  | Aux gas | 10 |
|  | Sweep gas | 0 |
|  | Probe heater temperature | 300 °C |
|  | S-lens RF level | 55 |
|  |  |  |
| PRM method | Collision energies | Long: 19% Total: 18% |
|  | Transitions | Long: y5+, y6+, y7+ Total: y11^2+^, y12^2+^, y13^2+^ |
|  | Isolation window | 3 m/z |
|  | Automatic gain control target value | 3 × 10^6^ |
|  | Maximum injection time | 300 ms |
|  | Resolution setting | 70 000 |

**Validation of SNAP-25 [Simoa]**

The partial assay validation of the SNAP-25 [Simoa] assay focused on parallelism, spike recovery, and LLOQ. For parallelism (Suppl. Table 3), CSF samples were analyzed diluted (2-fold, 4-fold, 8-fold, 16-fold and 32-fold). Parallelism results measured below the LLOQ were excluded from the calculations. For spike recovery, three CSF samples (x2 diluted) were each analyzed untreated (non-spiked) or ‘spiked’ with 125 pg/mL of recombinant SNAP-25 (Suppl. Table 4). LOD and LLOQ was calculated from the average signal of 16 blanks (mean AEB=0.015, std=0.02) plus 3 standard deviations (LOD=0.02 AEB) and 10 standard deviations (LLOQ=0.032 AEB), respectively. The SNAP-25 concentration was then interpolated from the calibration curve (i.e., the analytical LLOQ) and subsequently multiplied by the sample dilution (x4) factor to get a LLOQ of 16.1 pg/mL (Suppl. Table 5).

The following equation was used to calculate spike recovery:

% recovery = (Concentration of spiked sample) / (Concentration of non-spiked + concentration of spiked buffer) ×100

|  | **Dilution** | **Calc. Conc (pg/mL)** | **Corrected dilution (pg/mL)** | **CV (%)** |
| --- | --- | --- | --- | --- |
| **CSF 1** – Low SNAP-25 | 2 | 3.2 | 6.5 | 10.9% |
|  | 4 | 1.4 | 5.6 |  |
|  | 8 | 0.3 | 2.1 | <LLOQ^1^ |
|  | 16 | NA | NA | <LLOQ |
|  | 32 | NA | NA | <LLOQ |
| **CSF 2 –** Medium SNAP-25 | 2 | 27.8 | 55.6 | 11.7% |
|  | 4 | 16.6 | 66.6 |  |
|  | 8 | 9.2 | 73.7 |  |
|  | 16 | 4.8 | 76.2 |  |
|  | 32 | 2.1 | 68.7 |  |
| **CSF 3** – High SNAP-25 | 2 | 67.9 | 135.9 | 14.1% |
|  | 4 | 39.2 | 156.7 |  |
|  | 8 | 21.2 | 169.5 |  |
|  | 16 | 10.7 | 171.3 |  |
|  | 32 | 6.3 | 200.3 |  |

**Supplementary Table 3.** Parallelism (Simoa assay validation). ^1^Lower limit of quantification.

| Sample | Spike conc. (pg/mL) | Non-spiked conc (pg/mL) | Expected (pg/mL) | Recovery (%) |
| --- | --- | --- | --- | --- |
| CSF 1 – Low SNAP-25 | 125 | 3.3 | 128.3 | 104.3 |
| CSF 2 – Medium SNAP-25 | 125 | 27.8 | 152.8 | 88.4 |
| CSF 3 – High SNAP-25 | 125 | 68.0 | 193.0 | 91.6 |

**Supplementary Table 4.** Spike recovery (Simoa assay validation).

**Supplementary Table 5.** Lower limit of quantification (Simoa assay validation). ^1^AEB; ^2^Standard deviation; ^3^Lower limit of detection; ^4^Lower limit of quantification.

| Mean of blanks (AEB^1^) | 0.015 |
| --- | --- |
| Std^2^ | 0.0017 |
| LOD^3^ (AEB) | 0.02 |
| LLOQ^4^ (AEB) | 0.032 |
| LLOQ (pg/mL) | 16.1 |

**Validation of MS SNAP-25 assay ([Long] and [Total])**

The partial assay validation of the MS SNAP-25 assay focused on precision and measurement range. For precision (Suppl. Table 7 and 8), two CSF samples were aliquoted and stored at -80°C pending analysis on different occasions in replicates. For measurement range, recombinant SNAP-25 was spiked into a CSF sample in different concentrations in four replicates and variation between repeated measurements was measured on one occasion (Suppl. Table 9). LLOQ and ULOQ was then identified by the lowest and highest mean level, respectively, of which the CV was less than 20%.’

**Supplementary Table 6**. Precision raw data (MS assay validation)**.**

|  | **SNAP-25 [Total] (pmol/L)** | | | | | | |  | **SNAP-25 [Long] (pmol/L)** | | | | | | |
| --- | --- | --- | --- | --- | --- | --- | --- | --- | --- | --- | --- | --- | --- | --- | --- |
|  | *Replicate* | | | | | | |  | *Replicate* | | | | | | |
|  | *1* | *2* | *3* | *4* | *5* | *6* | *7* |  | *1* | *2* | *3* | *4* | *5* | *6* | *7* |
| **CSF 1** |  |  |  |  |  |  |  |  |  |  |  |  |  |  |  |
| Day 1 | 39 | 48 | 49 | 39 | 45 |  |  |  | 10 | 13 | 11 | 10 | 10 |  |  |
| Day 2 | 43 | 44 | 39 | 38 | 43 | 41 | 46 |  | 9.3 | 9.4 | 8.5 | 9.2 | 8.8 | 10 | 10 |
| Day 8 (batch 1) | 63 | 58 | 58 | 59 | 57 |  |  |  | 13 | 11 | 12 | 13 | 11 |  |  |
| Day 8 (batch 2) | 46 | 57 | 53 | 51 | 55 |  |  |  | 9.1 | 10 | 12 | 11 | 12 |  |  |
| Day 9 | 52 | 54 | 55 | 52 | 52 |  |  |  | 9.3 | 11 | 13 | 12 | 11 |  |  |
| Day 10 | 56 | 49 | 52 | 56 | 51 |  |  |  | 8.7 | 8.5 | 9.2 | 8.9 | 8.3 |  |  |
| **CSF 2** |  |  |  |  |  |  |  |  |  |  |  |  |  |  |  |
| Day 1 | 13 | 15 | 18 | 17 | 16 |  |  |  | 3.3 | 3.5 | 3.3 | 3.5 | 3.1 |  |  |
| Day 2 | 16 | 14 | 15 | 15 | 15 | 20 | 14 |  | 2.4 | 2.5 | 2.9 | 2.7 | 3 | 2.5 | 2.5 |
| Day 8 (batch 1) | 18 | 16 | 16 | 21 | 14 |  |  |  | 2.7 | 2.7 | 3.7 | 3.2 | 2.5 |  |  |
| Day 8 (batch 2) | 16 | 17 | 18 | 15 | 20 |  |  |  | 2.4 | 2.7 | 3.3 | 3 | 2.5 |  |  |
| Day 9 | 19 |  |  | 13 | 20 |  |  |  | 3.7 |  |  | 3.1 | 3.9 |  |  |
| Day 10 | 16 | 19 | 18 | 17 | 17 |  |  |  | 2.2 | 2.3 | 2.2 | 1.3 | 1.7 |  |  |
|  |  |  |  |  |  |  |  |  |  |  |  |  |  |  |  |

**Supplementary Table 7**. Precision calculation (MS assay validation).

|  | **Average concentration (pmol/L)** | |  | **Repeatability** | | |  | **Reproducability** | |
| --- | --- | --- | --- | --- | --- | --- | --- | --- | --- |
|  |  |  |  | **Std (pmol/L)** | | **CV (%)** |  | **Std (pmol/L)** | **CV (%)** |
|  | |  | | |  | | | | |
| **SNAP-25 [total]** |  | |  |  | |  |  |  |  |
| CSF 1 | 50 | |  | 3.2 | | 6.5 |  | 7.2 | 14 |
| CSF 2 | 17 | |  | 2.2 | | 13 |  | 2.2 | 13 |
|  | |  | | |  | | | | |
| **SNAP-25 [Long]** |  | |  |  | |  |  |  |  |
| CSF 1 | 10 | |  | 1 | | 10 |  | 1.5 | 14 |
| CSF 2 | 2.8 | |  | 0.4 | | 13 |  | 0.6 | 22 |
|  |  | |  |  | |  |  |  |  |

**Supplementary Table 8**. Measurement range (MS assay validation)**.**

| **SNAP-25 [Total]** | | | | | | |  | **SNAP-25 [Long]** | | | | | | |
| --- | --- | --- | --- | --- | --- | --- | --- | --- | --- | --- | --- | --- | --- | --- |
| Replicate | | | | Average (pmol/L) | Std (pmol/L) | CV (%) |  | Replicate | | | | Average (pmol/L) | Std (pmol/L) | CV (%) |
| 1 | 2 | 3 | 4 |  |  |  |  | 1 | 2 | 3 | 4 |  |  |  |
| 1436 | 1680 | 1732 |  | 1616 | 158 | 9.8 |  | 963.1 | 976.2 | 825.3 |  | 921.6 | 83.6 | 9.1 |
| 827.1 | 810.2 | 776.1 |  | 804.5 | 26 | 3.2 |  | 574.3 | 568.3 | 538.6 |  | 560.4 | 19.1 | 3.4 |
| 18 | 16 | 18 | 14.6 | 16.7 | 1.7 | 9.9 |  | 49 | 46.8 | 45.3 | 52.4 | 48.4 | 3.1 | 6.4 |
| 13.5 | 11.9 | 14 | 14 | 13.4 | 1 | 7.7 |  | 15.1 | 22.8 | 23.5 | 24 | 21.3 | 4.2 | 19.7 |
|  | | | | | | |  | 3.4 | 3.2 | 3.3 | 3.4 | 3.3 | 0.1 | 2.8 |
|  |  |  |  |  |  |  |  | 1.9 | 1.6 | 2.4 | 2.7 | 2.2 | 0.5 | 23.1 |

**Supplementary Table 9.** Associations of SNAP-25 with hippocampal volume, entorhinal cortex thickness and cortical thickness. ^1^Spearman’s rank correlation coefficient; ^2^95% confidence interval.

|  | SNAP-25 [Simoa] | |  | SNAP-25 [Total] | |  | SNAP-25 [Long] | |
| --- | --- | --- | --- | --- | --- | --- | --- | --- |
|  | r_s_^1^ (CI_95%_)^2^ | p-value |  | r_s_ (CI_95%_) | p-value |  | r_s_ (CI_95%_) | p-value |
| **Aβ+** |  |  |  |  |  |  |  |  |
| Hippocampal vol. | 0.00(-0.3863 - 0.3797) | 0.985 |  | -0.01(-0.3881 - 0.3779) | 0.976 |  | -0.12(-0.4802 - 0.2766) | 0.546 |
| Entorhinal thick. | -0.36(-0.6513 - 0.02965) | 0.062 |  | -0.19(-0.5373 - 0.2039) | 0.322 |  | -0.36(-0.6562 - 0.02110) | 0.056 |
| Cortical thick. | -0.11(-0.4705 - 0.2881) | 0.589 |  | -0.02(-0.3979 - 0.3680) | 0.930 |  | -0.16(-0.5099 - 0.2398) | 0.423 |
|  |  |  |  |  |  |  |  |  |
| **Aβ-** |  |  |  |  |  |  |  |  |
| Hippocampal vol. | -0.11(-0.4835 - 0.3041) | 0.605 |  | -0.16(-0.5230 - 0.2554) | 0.440 |  | -0.23(-0.5779 - 0.1802) | 0.250 |
| Entorhinal thick. | -0.07(-0.4566 - 0.3350) | 0.726 |  | -0.01(-0.4019 - 0.3932) | 0.980 |  | -0.01(-0.4082 - 0.3869) | 0.951 |
| Cortical thick. | 0.07(-0.3411 - 0.4512) | 0.751 |  | 0.02(-0.3769 - 0.4178) | 0.906 |  | 0.07(-0.3357 - 0.4561) | 0.729 |
|  |  |  |  |  |  |  |  |  |

**Supplementary Table 10.** Associations of SNAP-25 Simoa in plasma with P-tau_181_ and P-tau_231_. ^1^Spearman’s rank correlation coefficient; ^2^95% confidence interval.

| SNAP-25 [Simoa] | Neat | |  | 2-fold diluted | |
| --- | --- | --- | --- | --- | --- |
|  | r_s_^1^ (CI_95%_)^2^ | p-value |  | r_s_ (CI_95%_) | p-value |
| **Plasma** |  |  |  |  |  |
| P-tau_181_ | -0.01(-0.3779 - 0.3619) | 0.961 |  | 0.11 (-0.2682 - 0.4524) | 0.570 |
| P-tau_231_ | -0.02 (-0.3828 - 0.3569) | 0.937 |  | 0.17(-0.2100 - 0.5002) | 0.370 |
|  |  |  |  |  |  |
